# Opioid analgesia and the somatosensory memory of neonatal surgical injury in the adult rat

**DOI:** 10.1016/j.bja.2017.11.111

**Published:** 2018-02-01

**Authors:** O. Moriarty, L. Harrington, S. Beggs, S.M. Walker

**Affiliations:** 1 Developmental Neurosciences Programme (Pain Research), UCL Great Ormond Street Institute of Child Health, London, UK; 2 Neuroscience, Physiology and Pharmacology, University College London, London, UK; 3 Department of Anaesthesia and Pain Medicine, Great Ormond Street Hospital NHS Foundation Trust, London, UK

**Keywords:** analgesics, opioid, animals, newborn, pain, surgical incision

## Abstract

**Background:**

Nociceptive input during early development can produce somatosensory memory that influences future pain response. Hind-paw incision during the 1st postnatal week in the rat enhances re-incision hyperalgesia in adulthood. We now evaluate its modulation by neonatal analgesia.

**Methods:**

Neonatal rats [Postnatal Day 3 (P3)] received saline, intrathecal morphine 0.1 mg kg−1 (IT), subcutaneous morphine 1 mg kg^−1^ (SC), or sciatic levobupivacaine block (LA) before and after plantar hind-paw incision (three×2 hourly injections). Six weeks later, behavioural thresholds and electromyography (EMG) measures of re-incision hyperalgesia were compared with an age-matched adult-only incision (IN) group. Morphine effects on spontaneous (conditioned place preference) and evoked (EMG sensitivity) pain after adult incision were compared with prior neonatal incision and saline or morphine groups. The acute neonatal effects of incision and analgesia on behavioural hyperalgesia at P3 were also evaluated.

**Results:**

Adult re-incision hyperalgesia was not prevented by neonatal peri-incision morphine (saline, IT, and SC groups > IN; P<0.05–0.01). Neonatal sciatic block, but not morphine, prevented the enhanced re-incision reflex sensitivity in adulthood (LA < saline and morphine groups, P<0.01; LA vs IN, not significant). Morphine efficacy in adulthood was altered after morphine alone in the neonatal period, but not when administered with neonatal incision. Morphine prevented the acute incision-induced hyperalgesia in neonatal rats, but only sciatic block had a preventive analgesic effect at 24 h.

**Conclusions:**

Long-term effects after neonatal injury highlight the need for preventive strategies. Despite effective analgesia at the time of neonatal incision, morphine as a sole analgesic did not alter the somatosensory memory of early-life surgical injury.

**Editor's key points**

• Nociceptive input during early development can produce long-term somatosensory memory by alterations in structure and function of nociceptive pathways.
• The effects of neonatal analgesia on re-incision hyperalgesia were studied in a rat hind-paw incision model of neonatal surgery.
• Neonatal sciatic nerve block with bupivacaine, but not intrathecal or subcutaneous morphine, prevented the enhanced re-incision reflex sensitivity in adulthood.
• The benefits of morphine analgesia were limited to the period of administration in neonates, suggesting that alternative or multimodal approaches are necessary to prevent long-term somatosensory memory.

Early-life pain and stress influence health outcome and the risk of chronic pain in adulthood.^1^, ^2^ Increased nociceptive input during critical early developmental periods can produce long-term somatosensory memory encoded by alterations in structure and function of nociceptive pathways. Future noxious stimuli can then unmask enhanced sensitivity. Neonates requiring intensive care, particularly those born preterm, undergo large numbers of painful procedural interventions and surgery that influence neurodevelopmental outcome and alter somatosensory function in later life.^3^, ^4^, ^5^, ^6^ Whilst there is increasing awareness of the need for adequate analgesia to improve acute and long-term outcomes after neonatal surgery,^7^ the most effective regimen is not established.

Plantar hind-paw incision is an established model of surgical injury and produces acute hyperalgesia in juvenile and adult rodents.^8^ After initial incision in the 1st postnatal week, but not at older ages, the degree and duration of re-incision hyperalgesia in later life are enhanced when compared with animals without this prior experience.^9^ As neonatal surgery has an added impact on altered sensory function after extreme preterm birth,^4^ we have investigated hind-paw incision at a similar early developmental stage [Postnatal Day 3 (P3)] in the rodent.^10^ P3 incision triggers persistent alterations in spinal synaptic signalling and microglial reactivity and in descending modulation, which influence somatosensory thresholds and future injury response.^11^, ^12^, ^13^, ^14^ Whilst primary afferent blockade with local anaesthetic reduces some persistent effects, long-term modulation by neonatal opioid analgesia has not been evaluated after hind-paw incision.

This observational study in a rodent model investigated acute and long-term outcomes after P3 hind-paw incision with saline or equianalgesic doses of intrathecal or subcutaneous morphine. The primary outcome was the effect of neonatal morphine on the degree and duration of behavioural hyperalgesia after re-incision in adults, compared with an age-matched group undergoing adult-only incision. Secondary outcomes included baseline sensory thresholds and post-incision reflex sensitivity after morphine or local anaesthetic sciatic nerve block, morphine effects on incision-induced spontaneous and evoked pain in adults with prior neonatal morphine in the presence or absence of incision, and behavioural hyperalgesia at the time of neonatal incision to confirm the acute analgesic efficacy of the chosen morphine dose regime. As we have previously evaluated spinal neuronal and microglial responses after adult incision,^12^, ^13^ preliminary experiments evaluated these outcomes at the time of neonatal incision, and age-related changes in spinal mu opioid receptor (MOR) expression.

## Methods

The methods are briefly described with reference to our previous publications. Additional details and an Animal Research: Reporting In-vivo Experiments (ARRIVE) Guidelines^15^ checklist are included as Supplementary Material.

### Experimental animals

**Fig 1:**
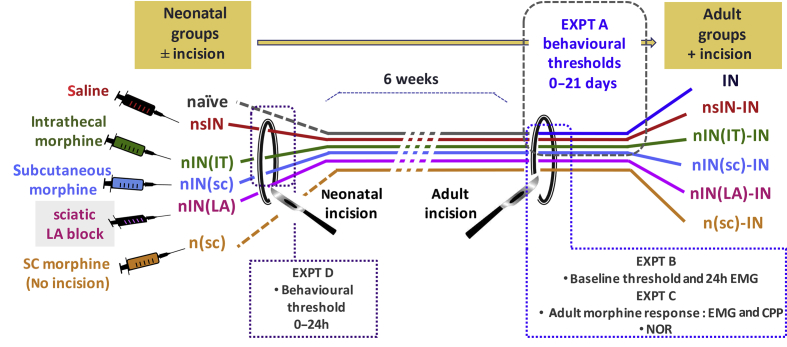
Schematic of experimental groups, timelines, and outcomes. Neonatal groups on Postnatal Day 3 had three × 2 hourly injections commencing 30 min before plantar hind-paw incision with saline, nsIN; intrathecal morphine 0.1 mg kg^−1^, nIN(IT); subcutaneous morphine 1.0 mg kg^−1^, nIN(SC); or sciatic nerve block with levobupivacaine 0.5%, nIN(LA). Additional groups received no treatment (naïve) or subcutaneous morphine alone, n(SC). The animals were returned to the dam (sutures were removed at 5 days), weaned into same-sex cages at 3 weeks, and were undisturbed until incision (IN) was performed at 6 weeks of age. Adult animals were assigned to Experimental (Expt) Groups A–C, and additional neonatal animals to Group D. CPP, conditioned place preference; EMG, electromyography; NOR, novel object recognition.

All experiments were performed under personal and project licenses in accordance with the UK Animals (Scientific Procedures) Act 1986. Sprague–Dawley rat pup litters or adult rats were obtained from the Biological Services Unit, University College London, London, UK. The handling and maternal separation of rat pups were kept to a minimum, and litters were weaned into same-sex cages at P21. Experimental groups comprised male and female rats distributed across multiple litters and adult cages. The rats were randomly selected, numbered, and allocated to treatment groups. The experimenters were blinded to treatment allocation during behavioural testing, electromyography (EMG) recording, video, or tissue analyses. Data are reported from 360 animals, and an additional 20 animals were used for pilot experiments. The experimental groups and timelines are detailed in Figure 1 and Supplementary Table S1.

### Plantar hind-paw incision

All surgery and injections were performed in male and female rats during isoflurane (Abbott, Maidenhead, UK) anaesthesia (2–4 vol% in 1 litres min^−1^ oxygen). The midline plantar hind-paw incision extended from the midpoint of the heel to the first footpad, with elevation and incision of the underlying plantaris muscle. Skin edges were closed with a single loop 5-0 silk suture in pups to produce stable knots, and two 5-0 mattress sutures in adult animals to standardise the model across all groups.^9^, ^12^ Neonatal incision was performed on the 3rd postnatal day (P3; body weight 9–13 g), and young adult incision at 6–7 weeks of age {body weight 195 [95% confidence interval (CI) 106, 265] g in males and 161 (95% CI 98, 201) g in females}.

### Behavioural testing

The rat pups were placed on a firm warming blanket to maintain body temperature. von Frey hair (vFh) filaments with increasing bending force (0.13–7.8 g) were sequentially applied five times to the dorsum of the hind paw and the number of evoked flexion reflexes recorded. The mechanical withdrawal threshold (50% effective force from sigmoidal stimulus–response curve)^9^ was measured before injections and 20 min later to confirm the drug effect (increase in threshold after morphine; unilateral motor block and no response to supra-threshold 13 g stimulus after sciatic block) before plantar incision.

Young adult rats were habituated to the test apparatus for measurements of mechanical withdrawal threshold (electronic von Frey device; Dynamic Plantar Aesthesiometer, Ugo Basile, Monvalle, Italy) and thermal withdrawal latency (University Anesthesia Research and Development Group, University of California, San Diego, CA, USA) at baseline and regular intervals to 21 days after adult incision. Threshold was designated as mean of three measures evoking a brisk withdrawal response.^12^, ^13^

### Electromyography

Flexor reflex EMG recordings were performed 24 h after adult incision.^9^, ^12^ The animals were anaesthetised with isoflurane, ventilated via a tracheal tube, and supported in a spinal frame. Isoflurane was maintained at 1.2 vol% for 20 min before and during recordings to allow mechanical ventilation without excessive reflex suppression. Data were included from 82 of 88 animals that had stable body temperature, heart rate, and oxygen saturation, whilst six were excluded because of unstable physiology and poor recording conditions. A bipolar EMG electrode in the biceps femoris recorded activity for 12 s after plantar hind-paw mechanical stimuli (vFh number 14–20, 13–120 g) (Neurolog; Digitimer, Welwyn Garden City, UK; PowerLab 4S; ADInstruments, Bella Vista, Australia). The integral of the EMG response was plotted against vFh number, and the area under the stimulus–response curve (AUC) quantified the overall ‘reflex response’.^9^, ^12^

### Drug administration

At P3, midline percutaneous lumbar intrathecal (IT) injections were performed^16^ with morphine 0.1 mg kg^−1^ or saline (injectate 0.5 μl g^−^^1^). Subcutaneous (SC) injection of 1 mg kg^−1^ morphine (5 μl g^−1^ of 0.2 mg ml^−1^) was performed in the same mid-lumbar site to ensure a separate investigator was blinded to injection route during testing. In anaesthetised adult animals, the EMG reflex sensitivity was quantified before (EMG AUC_1_) and 15 min after (EMG AUC_2_) SC morphine 0.75 mg kg^−1^ in the contralateral hindlimb [%baseline = (EMG AUC_2_/EMG AUC_1_) × 100] (see Supplementary Text S1 for dose finding). Percutaneous sciatic block with levobupivacaine 0.5%, 40 μl (Chirocaine; Abbott Laboratories Limited, Maidenhead, UK) was performed before incision and at two × 2 hourly intervals at P3.^9^, ^14^ Effective block (ipsilateral motor block and loss of withdrawal to supra-threshold 13 g vFh) was confirmed before incision.

### Conditioned place preference

The following sequence was used for conditioned place preference (CPP) in adult males^17^: (1) preconditioning day (D1), placed in a central connecting chamber and ‘preferred’ of two end chambers with different visual cues noted, and then hind-paw incised; (2) D2, single-trial biased-design conditioning with SC saline and placed in the preferred chamber for 45 min, then 4 h later SC morphine (2 mg kg^−1^) and placed in the non-preferred chamber; and (3) D3, placed in the central chamber with free access and the time spent in each chamber measured over 15 min (schematic of the test apparatus included in figure). Data are expressed as total time spent in the initially non-preferred chamber during pre-conditioning *vs* during the test session, or a relative difference score (positive score demonstrates preference for morphine-paired chamber).^17^

### Novel object recognition

After habituation to the empty test arena, adult incision was performed the following day. Testing 24 h later comprised habituation (arena for 3 min and return to home cage for 7 min); exposure 1 (two identical objects in arena, freely explore for 3 min, home cage for 10 min); and exposure 2 [one familiar object replaced by the novel object, free exploration for 3 min (schematic of the test apparatus included in figure)]. The duration of object exploration within a 2 cm annulus (sniffing, rearing against, or having the head directed towards the object) was timed manually from video recordings (discrimination ratio = total time spent exploring either object/total time spent exploring both objects).^18^, ^19^

### Tissue analysis

Preliminary spinal tissue analyses are included in the Supplementary Materials. Immunohistochemistry of lumbar spinal cord (L4/L5) segments assessed c-Fos immunohistochemistry 2 h after P3 incision, ionised calcium-binding adapter molecule 1 (Iba1) 3 days after neonatal incision, and MOR distribution at P3 and P42.

### Statistical analysis

The primary outcome was the effect of neonatal peri-incision morphine on adult re-incision hyperalgesia. The hyperalgesic index for each animal was calculated as the area over the percentage change in sensory threshold *vs* the time curve from baseline to 21 days (AOC 0–21 days),^20^ and so higher values represent an increased degree and duration of hyperalgesia. Based on our previous data using this methodology in rats from the same colony, a sample size of eight has 90% power for detecting a 30% difference (*P*<0.05) in mechanical hyperalgesic index.^12^, ^13^ For raw sensory thresholds, a sample size of eight has 80% power at *P*<0.01 for detecting a 20–25% difference in mechanical withdrawal threshold in adult males and females.^14^

Normally distributed data (D'Agostino and Pearson test) were analysed by unpaired Student's *t*-test or one-way analysis of variance (anova) to assess group differences, two-way anova with sex and treatment group as variables, or factorial anova with sex and treatment group as between-subject factors and repeated measures of time for behavioural thresholds. Tukey *post hoc* tests or Dunnett's comparison to baseline were used with *P*-values adjusted for multiple comparisons (SPSS Statistics Version 23, IBM, Portsmouth, UK; Prism Version 7, GraphPad, San Diego, CA, USA). *P*<0.05 was considered statistically significant.

## Results

### Morphine at the time of neonatal incision does not prevent enhanced re-incision hyperalgesia in adulthood

**Fig 2:**
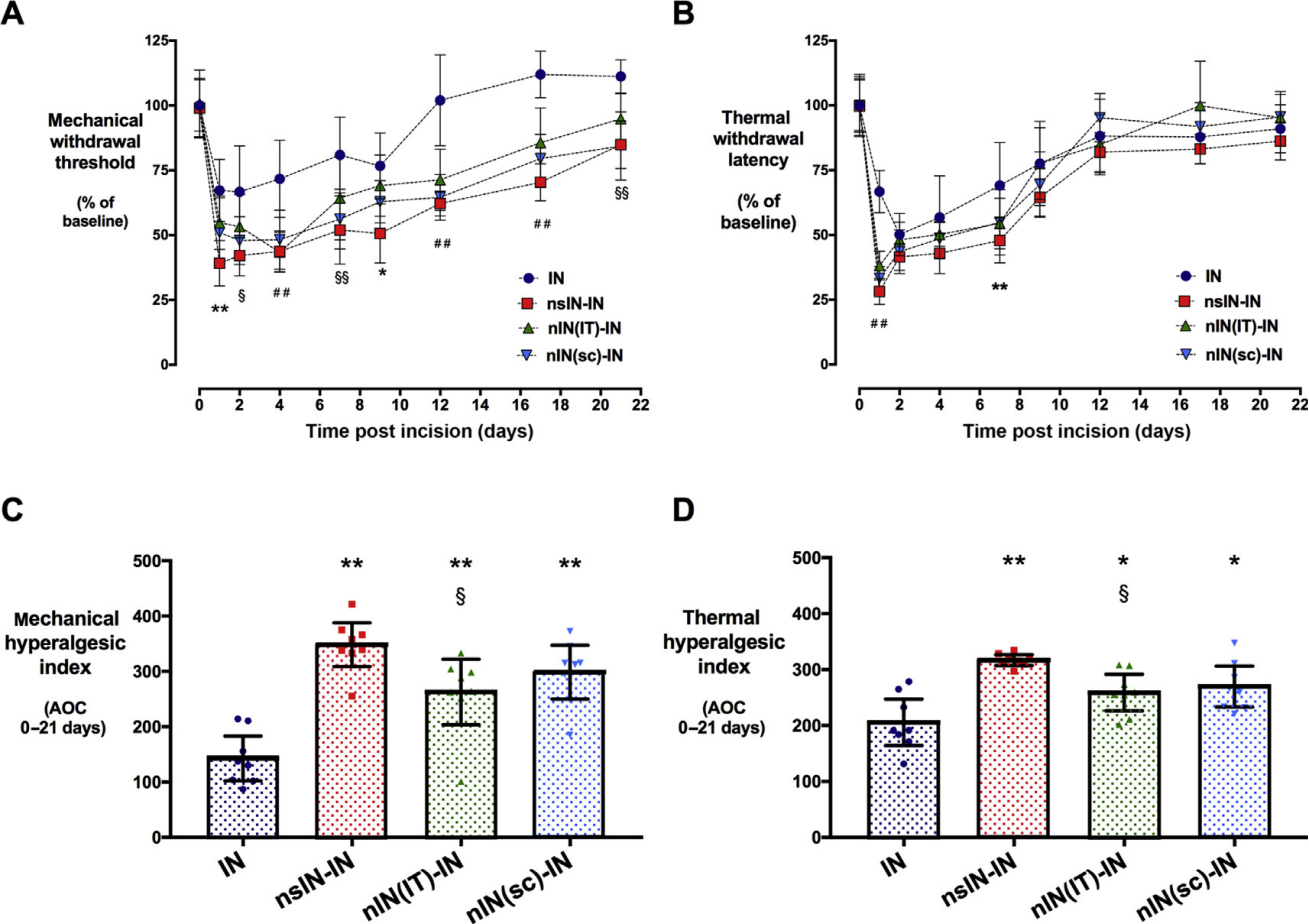
Neonatal peri-incision morphine does not prevent re-incision hyperalgesia in adult animals. (a) Mechanical withdrawal threshold and (b) thermal withdrawal latency of the left hind-paw as percentage change from pre-incision baseline (100%) at time points to 21 days post adult incision (IN) or prior neonatal incision with saline (nsIN-IN), intrathecal morphine [nIN(IT)-IN], or subcutaneous morphine [nIN(SC)-IN]. Data points = mean (95% confidence interval), *n*=8 per group; *P<0.05, **P<0.01 IN *vs* nsIN-IN; ^##^*P*<0.01 IN *vs* nsIN-IN, nIN(IT)-IN, nIT(SC)-IN; ^§§^*P*<0.01 IN *vs* nsIN-IN and nIN(SC)-IN; two-way repeated measures anova with Tukey between group *post hoc* comparisons. (c,d) Hyperalgesic index calculated as area over the threshold *vs* time graph for mechanical withdrawal threshold and thermal withdrawal latency. Individual data points, bars = mean [95% confidence interval]; *n*=8 per group. *P<0.05, **P<0.01 *vs* IN; ^§^*P*<0.05 nsIN-IN *vs* nIN(IT)-IN; one way anova with Tukey *post hoc* comparisons. anova, analysis of variance.

As reported previously,^12^, ^13^ prior neonatal incision enhanced re-incision hyperalgesia in adult rats (Fig. 2). The behavioural thresholds for males and females are combined and presented as percentage change to account for differences in baseline threshold (raw data for incised and contralateral paws in Supplementary Fig. S1). After incision, the reduction in mechanical threshold was greater in re-incision groups [main effect of group *F*_3,28_=22, *P*<0.001; IN *vs* nsIN-IN, nIN(IT)-IN, nIN(sc)-IN, all *P*<0.01] (Fig. 2a). Mechanical hyperalgesia was prolonged in prior neonatal incision groups [IN 9 days; nsIN-IN 17 days; nIN(IT)-IN 12 days; nIN(sc)-IN to 17 days; two-way anova Dunnett's comparison with baseline]. Thermal hyperalgesia was similarly prolonged after re-incision (IN 9 days; nsIN-IN 17 days), but differences in degree were less marked (main effect of group *F*_3,28_=6.5, *P*=0.002; IN *vs* nsIN-IN *P*<0.001), and morphine groups differed from IN only at 24 h (Fig. 2b).

The hyperalgesic index (i.e. area over the threshold *vs* time curve) incorporates differences in both the degree and duration of hyperalgesia. The mechanical hyperalgesic index was increased by prior neonatal incision (main effect of group *F*_3,28_=19; *P*<0.001), and not altered by co-administration of morphine (IN *vs* all other groups; *P*<0.01; Fig. 2c). The thermal hyperalgesic index increased after prior incision (main effect of group *F*_3,28_=11; *P*<0.001), with or without neonatal morphine (Fig. 2d). Intrathecal morphine had a partial modulatory effect [nIN(IT)-IN < nsIN-IN; *P*<0.05], but did not prevent the enhanced incision response [nIN(IT)-IN > IN; *P*<0.05] (Fig. 2c and d).

### Neonatal peri-operative sciatic blockade, but not morphine, prevents alterations in adult spinal reflex sensitivity

**Fig 3:**
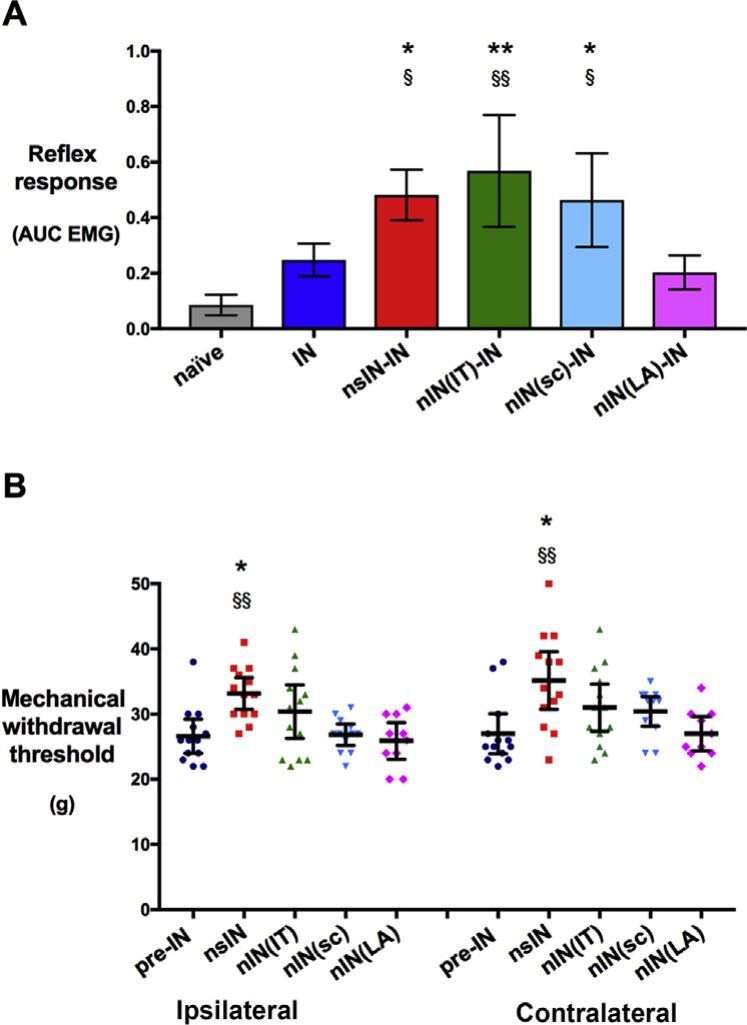
Neonatal sciatic block more effectively reduces re-incision hyperalgesia than morphine. (a) Reflex sensitivity [area under mechanical stimulus *vs* biceps electromyography response curve (AUC EMG)] 24 h after incision was in prior neonatal incision with saline (nsIN-IN), intrathecal morphine (nIN(IT)-IN), or subcutaneous morphine (nIN(SC)-IN) when compared with IN (*P<0.05, **P<0.01 *vs* IN) or sciatic block groups [^§^*P*<0.05, ^§§^*P*<0.01 *vs* nIN(LA)-IN]. Bars = mean [95% confidence interval]; *n*=10–13 per group; one-way anova with Tukey *post hoc* comparisons. (b) Baseline mechanical withdrawal thresholds before adult incision were higher in both the ipsilateral and contralateral paw of animals with prior neonatal incision (*P<0.05 *vs* IN), and this effect was blocked by neonatal sciatic local anaesthetic block [^§§^*P*<0.01 *vs* nIN(LA)]. Individual data points shown; bars = mean [95% confidence interval]; *n*=10–13 per group; two-way anova with Tukey *post hoc* comparisons. anova, analysis of variance; AUC, area under the stimulus–response curve; EMG, electromyography.

Reflex EMG sensitivity 24 h after adult incision demonstrated a main effect of group (*F*_4,51_=6.1; *P*<0.001), but not sex (*F*_1,51_=0.1; *P*=0.8), with increased hyperalgesia after prior neonatal incision (nsIN-IN > IN; *P*=0.04). The neonatal morphine groups did not differ from nsIN-IN, whereas sciatic nerve block prevented the enhanced re-incision response [nIN(LA)-IN < nsIN-IN; *P*=0.02] (Fig. 3a).

Before incision and EMG recordings, the baseline mechanical withdrawal thresholds of the left hind paw showed a main effect of group (*F*_4,51_=4.7; *P*=0.003), but not sex (*F*_1,51_=1.5; *P*=0.23), with higher thresholds after neonatal incision (nsIN > naive; *P*=0.02). This effect was generalised to both hind paws, but was prevented by neonatal sciatic block [nIN(LA) < nsIN; *P*<0.01]. Baseline thresholds after neonatal incision with either intrathecal or subcutaneous morphine did not differ from other groups (Fig. 3b).

### Morphine analgesia in adulthood is not altered when morphine is co-administered with neonatal incision

**Fig 4:**
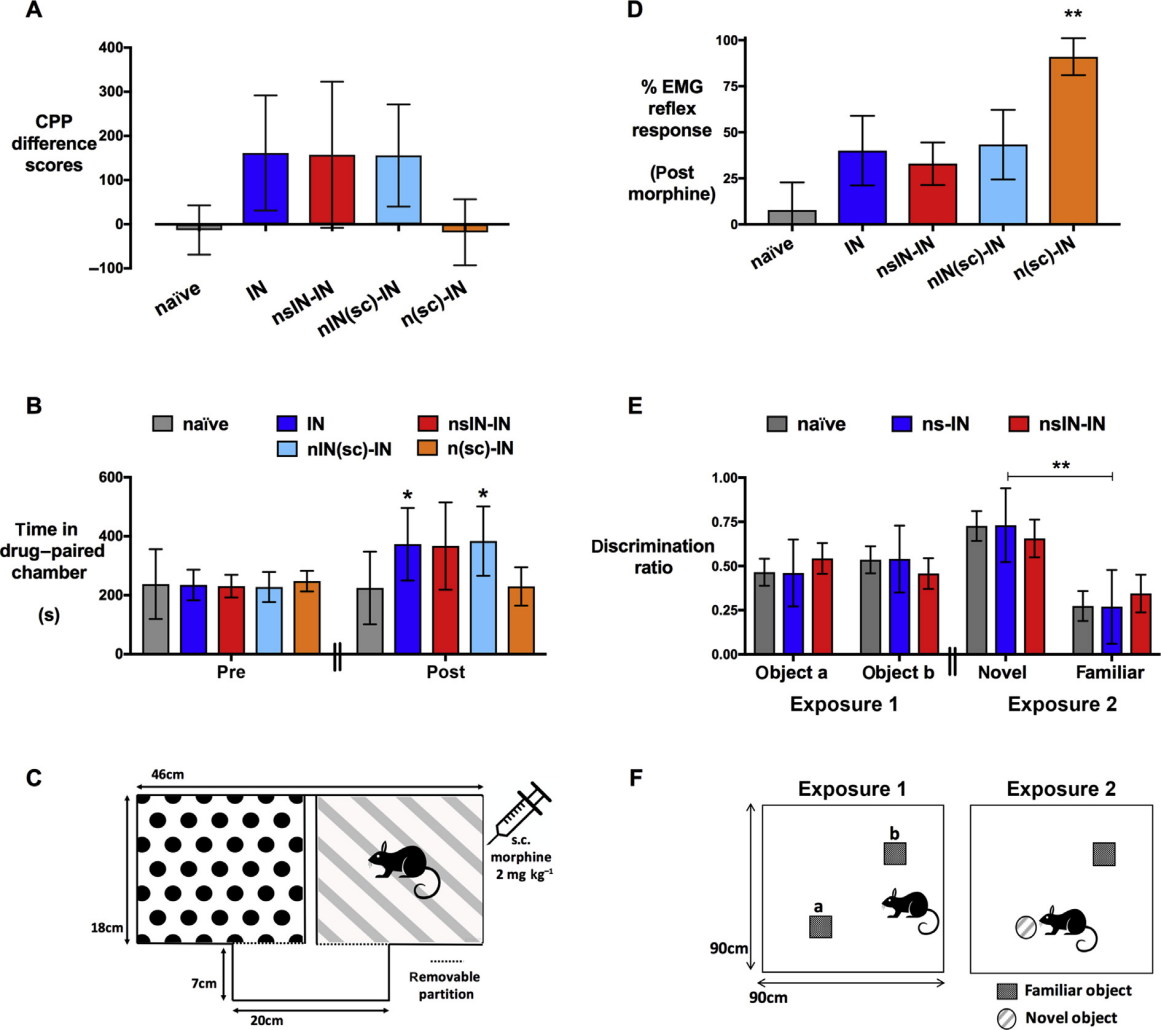
Adult morphine response differs if prior neonatal morphine is given in the presence or absence of neonatal incision. (a) CPP demonstrates positive difference scores (time spent in morphine-paired chamber during the test session minus the time spent in that chamber during pre-conditioning) when neonatal morphine was given at the time of incision, but not when given alone [nIN(SC)-IN *vs* n(SC)-IN; *P*<0.05, two-tailed Student's *t*-test]. (b) Time spent in the morphine-paired chamber increased after the conditioning trial in nIN(SC)-IN, but not n(SC)-IN groups. Bars = mean [95% confidence interval]; *n*=8–12 per group. (c) Schematic of CPP apparatus and chambers with differing visual cues. The initial preferred chamber is identified with access to both chambers during preconditioning. Biased design conditioning includes injection in the initial preferred chamber with the partition closed, and morphine in the alternate chamber 4 h later. Preference for the morphine-paired chamber is subsequently assessed with access to both chambers. (d) Percentage change in post-incision reflex sensitivity (quantified from the area under the mechanical stimulus *vs* electromyography response curve, AUC EMG) by 0.75 mg kg^−1^ subcutaneous morphine was less in the n(SC)-IN group. Bars = mean [95% CI]; *n*=12–13 per group. **P<0.01 n(SC)IN-IN *vs* all other groups, one-way anova with Tukey *post hoc* comparisons. (e) Adult rats spent similar time exploring Objects a and b at baseline (Exposure 1), but increased time exploring the novel object in Exposure 2 (discrimination ratio >0.5). This did not differ in incised adults either with prior neonatal anaesthesia (ns-IN) or neonatal anaesthesia and surgery (nsIN-IN). Bars = mean [95% CI]; naive, *n*=14; nsIN, *n*=6; nsIN-IN, *n*=22. **P<0.01 familiar *vs* novel for all treatment groups with two-way anova and Tukey *post hoc* comparisons. (f) Schematic of open field and object placement. anova, analysis of variance; AUC, area under the stimulus–response curve; CI, confidence interval; CPP, conditioned place preference; EMG, electromyography.

At 24 h after adult incision, we compared the effects of morphine on spontaneous pain (CPP) and evoked pain (EMG reflex sensitivity) in animals with prior neonatal incision and SC morphine (Fig. 4). After adult incision, positive CPP difference scores were seen in IN, nsIN-IN, and nIN(SC)-IN groups, whereas non-incised (naive) animals did not show a preference for the morphine-paired chamber (Fig. 4a). Neonatal morphine had differing effects if given alone or in combination with neonatal incision. At baseline, there were no significant group differences, but the time spent in the drug-paired chamber increased after morphine conditioning in the nIN(sc)-IN group, but not the n(SC)-IN group (Fig. 4b and c).

Morphine 0.75 mg kg^−1^ SC reduced reflex sensitivity to 30–40% of baseline after single adult incision (IN), and this was not altered by prior neonatal incision with or without neonatal morphine (Fig. 4d). However, in animals that received neonatal morphine in the absence of tissue injury, morphine had less effect after adult incision, with reflex sensitivity remaining at 88% (95% CI 68, 107) and 94% (95% CI 81, 107) of baseline in males and females, respectively (*n*=6 per group). Neonatal morphine alone did not alter the baseline mechanical withdrawal threshold in adulthood [n(SC)-IN *vs* IN, 29.6 (95% CI 25, 34) *vs* 29.6 (95% CI 28, 31) g] and had a minor effect on thermal withdrawal latency [8.9 (95% CI 7.8, 10.0) *vs* 10.3 (95% CI 9.7, 11) s; *P*=0.048, Student's unpaired *t*-test].

The potential effects of neonatal incision and repeated anaesthesia on recognition memory in adulthood were tested with novel object recognition. Movement in the open field (data not shown) and time spent exploring objects did not differ between naïve and incision groups at baseline (Fig. 4e and f). In the subsequent exposure, all groups spent more time near the novel object, including those exposed to repeated neonatal anaesthesia for saline injections or neonatal anaesthesia plus incision (*P*<0.001; Fig. 4e). There was no main effect of group (*F*_2,36_=0.7; *P*=0.52) or sex (*F*_1,36_=0.6; *P*=0.45) on time spent with the novel object.

### Perioperative morphine modulates acute hyperalgesia after neonatal incision, but the effects are limited to period of administration

**Fig 5:**
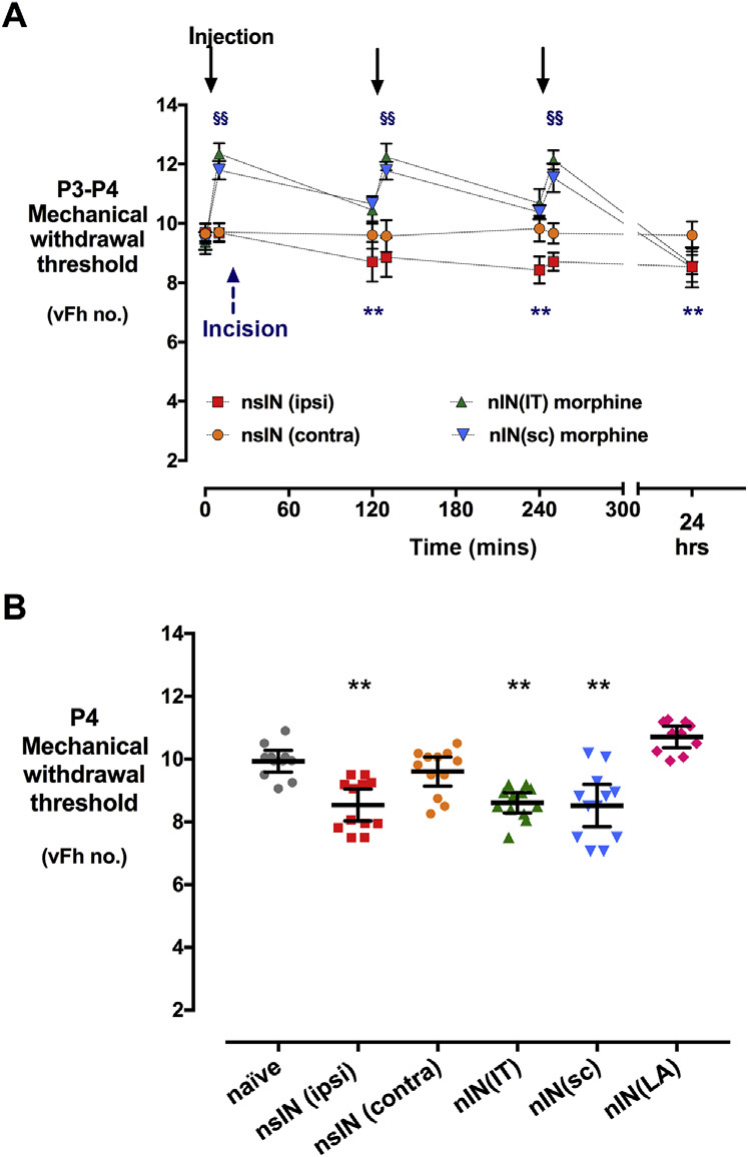
Acute effects of neonatal incision and analgesia on behavioural hyperalgesia. (a) Mechanical withdrawal threshold at baseline (time =0) on Postnatal Day 3 and after injections and incision. Incision produced hyperalgesia [**P<0.01, nsIN (neonatal saline, incision ipsilateral hind-paw) < nsIN (contralateral un-incised)]. Intrathecal morphine 0.1 mg kg^−1^ [nIN(IT) morphine] and subcutaneous morphine 1 mg kg^−1^ [nIN(SC) morphine] increased the threshold to a similar degree, and maintained threshold above baseline at 2 and 4 h, but not 24 h [§§*P*<0.01 *vs* nsIN (contra)]. Data points = mean [95% CI]; *n*=12 per group; two-way repeated measures anova with Tukey *post hoc* comparisons. (b) Mechanical withdrawal threshold on P4. Peri-incision sciatic block [(nIN(LA), *n*=10] prevents hyperalgesia at 24 h. Individual data points, bars = mean [95% CI]; **P<0.01 nsIN(ipsi), nIN(IT), and nIN(SC) *vs* naïve, nsIN(contra), and nIN(LA). CI, confidence interval.

Neonatal P3 incision acutely reduced the mechanical withdrawal threshold (incised *vs* contralateral paw **P<0.01) (Fig. 5a). Morphine IT 0.1 mg kg^−1^ or SC 1 mg kg^−1^ (three × 2 hourly injections) was equianalgesic and maintained the threshold above baseline for 6 h. By 24 h, the mechanical withdrawal threshold in the morphine groups did not differ from saline incised controls, whereas a reduction in hyperalgesia was still seen (i.e. preventive analgesic effect extending beyond the duration of drug effect) after sciatic block (Fig. 5b). Weight gain was observed in all groups (range, 9–13 g at P3; 11–15 g at P4).

Preliminary tissue analyses demonstrated that sciatic block was more effective than morphine at preventing incision-induced c-Fos activation in superficial laminae of the dorsal horn, but neither morphine nor sciatic block altered the Iba1 cell counts in the medial superficial dorsal horn 3 days after neonatal incision (Supplementary Fig. S2). Systemic morphine at P3 did not alter age-dependent changes in spinal MOR immunoreactivity (broad dorsal horn distribution at P3, but restriction to superficial laminae I and II at P45) (Supplementary Fig. S3).

## Discussion

Neonatal incision produces somatosensory memory that influences future injury response. Morphine by either systemic or intrathecal routes controlled acute incision-induced hyperalgesia in neonatal animals, but did not prevent enhanced behavioural allodynia or hyperalgesia after re-incision in later life. In contrast, neonatal peri-incision sciatic nerve block prevented alterations in adult baseline sensory thresholds and spinal reflex sensitivity after re-incision. Morphine efficacy in adulthood was not altered after neonatal incision and morphine, but was reduced when neonatal morphine was administered in the absence of tissue injury. These data highlight the need to evaluate the impact of different analgesic regimens on both acute and persistent effects of surgical injury to identify potential preventive interventions. Despite being an effective analgesic during the neonatal period, morphine as a sole analgesic did not alter the somatosensory memory of early-life incision.

The impact of prior neonatal pain and tissue injury on somatosensory function in adulthood has been identified in many preclinical injury models.^2^ We have used plantar hind-paw incision as a clinically relevant model of surgical injury, as damage to skin and peripheral nerves and local inflammation can all contribute to postoperative pain,^21^ but responses to these individual components vary with postnatal age and severity of injury.^2^ In line with our previous studies,^12^, ^13^ the degree and duration of behavioural allodynia and reflex hyperalgesia were enhanced after adult re-incision. Whereas neonatal hind-paw inflammation with complete Freund's adjuvant can produce chronic inflammation,^22^ 0.25% carrageenan produces a similar pattern as incision (i.e. brief acute hyperalgesia in neonatal rodents, but enhanced hyperalgesia after repeat inflammation^23^ or hind-paw incision^24^ in adulthood). Effects are specific to an initial injury in the 1st postnatal week, suggesting a critical period when altered activity in the developing nervous system triggers persistent changes in function.^9^

Modulation of this somatosensory memory by different types and routes of analgesic intervention has not been widely studied. Morphine at the time of neonatal carrageenan inflammation reduced re-inflammation hyperalgesia in adult females and to a lesser degree in males,^25^ and we found partial reduction in adult behavioural allodynia, but not reflex hyperalgesia after neonatal incision with intrathecal morphine. Although intrathecal or subcutaneous morphine effectively blocked acute hyperalgesia at the time of neonatal incision, enhanced response to re-incision was still observed in adulthood. We previously reported that sciatic block at the time of neonatal incision prevented enhanced EMG reflex sensitivity 24 h after re-incision 2 weeks later,^9^ and our current results confirm this benefit extends into adulthood. As we have demonstrated consistent re-incision group differences with both repeated behavioural thresholds to 4 weeks, and EMG measures of reflex sensitivity at 24 h, and 1 and 2 weeks after adult incision, the analysis after sciatic block was restricted to a single time point. Differences between the neonatal sciatic block and morphine groups demonstrate the sensitivity of our model for comparing analgesic effects on somatosensory memory.

Prior neonatal hind-paw carrageenan inflammation^23^ or incision^14^ has dual effects on somatosensory function that differ in time course and distribution. Re-injury hyperalgesia can be evoked after 1–2 weeks and is segmentally restricted, whereas raised baseline sensory thresholds emerge after the 4th postnatal week and have a generalised distribution associated with altered descending modulation from the rostroventral medulla (RVM).^14^, ^26^ In addition to normalising the adult balance of descending inhibition and facilitation from the RVM,^14^ neonatal peri-incision sciatic block also prevents generalised hypoalgesia in adulthood. Reported effects of neonatal morphine on adult sensory threshold vary with injury model and dose. On the day of birth (P0), administering morphine with carrageenan inflammation^25^ or abdominal incision^27^ normalised the sensory thresholds in adult rodents. Alterations in adult hot-plate latency after repeated neonatal hind-paw formalin were ‘partially ameliorated’ by morphine in males but not in females.^28^ Here, morphine had some impact on adult sensory thresholds, but the differences did not reach the degree seen with sciatic block. Sex differences after neonatal interventions vary with type of injury, analgesia, and subsequent outcome.^29^ As previously reported in adult Sprague–Dawley rats,^30^ we found similar responses to incision and morphine in males and females.

The impact of neonatal exposure to morphine and injury on opioid efficacy in adulthood differs across studies, and doses vary widely if the aim is to model neonatal analgesia or tolerance/withdrawal. Neonatal hind-paw incision^31^ did not alter the anti-nociceptive effects of morphine on thermal latency in young adult rodents, and here, prior neonatal incision did not alter the anti-hyperalgesic efficacy of morphine after adult re-incision. However, prior neonatal exposure to morphine alone has been associated with reduced opioid efficacy in later life,^32^, ^33^ and the pattern differs from ‘tolerance’ as it occurs after several weeks, is apparent on the first subsequent dose, and may be specific to exposure at younger ages.^32^, ^34^

Direct comparisons suggest that effects differ when morphine is given alone or in combination with a painful injury. The ED_50_ of morphine in adult rats was altered after neonatal inflammation, but not after morphine alone or combined inflammation and morphine.^25^ Conversely, neonatal morphine alone produced a marked right shift in subsequent dose–response, but when given in conjunction with hind-paw inflammation the adult efficacy did not differ from controls.^35^ Similarly, we found that the analgesic response to morphine in adult tests of both evoked and spontaneous pain was maintained when neonatal morphine was administered with incision, but reduced when exposure to neonatal morphine occurred in the absence of injury. Whilst systemic morphine at P3 did not alter the previously documented age-related changes in spinal MOR distribution,^36^ the magnitude and mechanisms of persistent alterations in opioid efficacy require further evaluation.

CPP assesses the motivational drive to seek relief from ongoing or spontaneous pain.^37^ At 24 h after hind-paw incision, single-trial conditioning with peripheral local anaesthetic blocks induced preference for the analgesia-paired chamber.^17^, ^37^ Morphine-induced CPP effects vary with dose, presence or type of injury, and time. At 14 days after thoracotomy in adult rodents, gabapentin but not morphine induced CPP,^38^ possibly because of the emergence of neuropathic pain that shows variable CPP to morphine.^39^ Whilst neonatal incision alone did not alter morphine CPP in uninjured adolescent rats,^31^ long-term alterations have been seen after repeated neonatal stress or morphine alone, but not when both are combined.^40^, ^41^ Here, preference for the morphine-paired chamber was evident after incision. Despite differences in the degree of hyperalgesia, CPP did not differ between single and re-incision groups. The increased reflex sensitivity might not be associated with a similar increase in the degree of aversiveness (i.e. there is no further increase in the motivational drive to relieve pain mediated by central reward circuits), or subtle differences may have been obscured by the variability in this outcome. Importantly, whilst CPP was not altered when neonatal morphine was administered with incision, animals exposed to neonatal morphine in the absence of injury failed to show a clear preference for the morphine-paired chamber, suggesting reduced opioid efficacy in this group. These data further highlight the need to evaluate potential modifying effects of surgical injury alongside long-term effects of either anaesthesia or analgesia. Early-life anaesthesia has been associated with impaired recognition memory in children and rodents.^42^ Novel object testing exploits rats' natural tendency to explore novel rather than familiar stimuli, and evaluates non-hippocampal-dependent learning and memory.^43^ Novel object recognition was not impaired after the brief but repeated exposures to anaesthesia for control saline injections either alone, or in combination with surgical injury, on P3.

Dose requirements for systemic,^44^ epidural,^45^ and intrathecal^16^ morphine are influenced by postnatal age and are lower in P3 pups. Doses of systemic and intrathecal morphine were equi-analgesic and sufficient to prevent acute incision-induced hyperalgesia during the period of administration, but did not have the preventive analgesic effect at 24 h seen with sciatic nerve block. Morphine was also less effective than sciatic block at suppressing neuronal activation (c-Fos immunoreactivity) in the superficial dorsal horn. Whilst morphine influences spinal microglial reactivity in adults^46^ and neonatal incision primes the spinal microglial response to re-incision,^12^, ^13^ neither morphine nor sciatic block altered microglial reactivity 3 days after neonatal incision.

The strengths of this study include the use of an established surgical injury model to demonstrate a somatosensory memory of prior neonatal incision. Opioids are common perioperative analgesics for neonates,^47^ and we titrated morphine to block acute hyperalgesia after neonatal hind-paw incision. Whilst subsequent failure to prevent re-incision hyperalgesia was confirmed after systemic or intrathecal administration, we cannot exclude a potential benefit from more prolonged dosing in the neonatal period. Sciatic nerve block served as a positive control, and demonstrated the sensitivity of our model for comparing both the acute and long-term impacts of different analgesic interventions. Only limited conclusions can be drawn regarding alterations in opioid efficacy after neonatal exposure, which are in line with previous studies, but require further evaluation. There were no main effects of sex for the assessed outcomes, although smaller differences may be identified with larger sample sizes. We tested morphine CPP in males only, but no sex differences have previously been reported for this outcome in adult Sprague–Dawley rats.^48^ To minimise the number of animals used, secondary outcomes were assessed in the most relevant rather than all possible treatment groups. Whilst our observational studies demonstrate clearly the failure of morphine to alter the somatosensory memory of neonatal incision, additional investigations are required to delineate underlying mechanisms.

Our preclinical studies demonstrate somatosensory memory of early-life surgical injury. Comparison of the same injury at different postnatal ages confirms a specific developmental effect. Clinically, repeat surgery has been shown to increase pain and analgesic requirements in infants,^49^ and increases the risk of persistent post-surgical pain in adults.^50^ However, it is difficult to identify a clinical critical period when surgery has an added impact on nociceptive processing, as the types of surgery vary with age and persistent post-surgical pain is also influenced by factors, such as pre-existing pain, psychological factors, and pain-coping style.^50^ However, there is clear clinical evidence of the increased vulnerability of the developing nervous system to surgery and anaesthesia in early life,^51^ and neonatal surgery has an added impact on long-term neurodevelopmental outcome and somatosensory function after preterm birth.^2^, ^4^, ^5^, ^52^ Therefore, we suggest that significant early-life pain exposure during neonatal intensive care and surgery should be considered when planning perioperative care in later life.

Optimal peri-operative analgesia requires titration against individual response, consideration of the type of surgery, and evaluation of the relative risks and benefits of different drugs and techniques.^53^ Multimodal peri-operative analgesia regimens are recommended for children^47^ and adults,^50^ but the appropriate combination for specific procedures^53^ and longer-term effects on persistent post-surgical pain or repeat surgery response have not been evaluated.^54^ The current studies align with clinical data demonstrating dose-dependent efficacy of morphine for acute perioperative analgesia. However, the benefits from morphine as a sole analgesic were limited to the period of administration in neonatal rodents, and in adult were influenced by prior neonatal experience. Evaluating the comparative efficacy of different analgesic interventions in standardised preclinical models can provide important data to inform the design of clinical trials aiming to improve both acute and long-term outcomes.

## Authors' contributions

Study design/planning: O.M., S.B., S.M.W.

Study conduct: O.M., L.H., S.M.W.

Data analysis: O.M., L.H., S.M.W.

Drafting/writing paper: S.M.W.

Revising/approving paper: all authors.

## Appendix A Supplementary data

mmc1

mmc2

mmc3

mmc4

mmc5

mmc6
