# Supplementary material for: Opioid analgesia and the somatosensory memory of neonatal surgical injury in the adult rat
Source: Br J Anaesth. 2018 Feb 1;121(1):314–24. doi: 10.1016/j.bja.2017.11.111 (PMC6200106; doi:10.1016/j.bja.2017.11.111)
Supplement: mmc1 [file mmc1.docx]

**Supplementary Text 1. Additional Details for Methods, Dose-finding Experiments, and Preliminary Tissue Analyses.**

**Experimental animals**

# Sprague-Dawley rat pup litters or adult rats from the Biological Services Unit University College London were bred and maintained in-house and exposed to the same caging, handling and diet throughout development. Animals were maintained on a 12-h light/dark cycle at constant ambient temperature with free access to food and water. For rat pups, body temperature was maintained on a heating blanket during procedures and recovery. Pups were weaned into same-sex cages (4-5 per cage) at 3 weeks, and apart from routine care and regular monitoring by Biological Services Unit staff and experimenters, were left undisturbed until early adulthood. Experiments requiring surgery or repeated injections commenced in the morning to allow time for recovery and monitoring at the end of the procedures, prior to animals being returned to the Biological Services Unit. On testing or experimental days, animals were transferred to Home Office approved laboratories in an adjacent building at UCL. Experiments requiring repeated behavioural assessments on multiple days were performed as close as possible to the same time in the morning to allow time for habituation to the test apparatus.

**Plantar hindpaw incision**

Following application of alcoholic chlorhexidine gluconate 0.5% (Vetasept, Animalcare Ltd, York, UK) to the plantar aspect of the left hindpaw, a midline incision through the skin and fascia was made and the underlying plantaris muscle was elevated and incised longitudinally.^1^ The same relative length of incision was performed in neonatal and adult animals, extending from the midpoint of the heel to the first footpad.^2, 3^ Skin edges were closed with a single loop 5-0 silk sutures (Ethicon, Edinburgh, UK) in pups. In the small hindpaw this suture material could be tied easily without pulling through the fragile skin edges and was sufficiently stable when close cut to the skin to avoid disruption and removal of foreign material by the dam. To standardize the model, we also used 5-0 silk with two mattress sutures in adult animals (rather than Nylon sutures as originally described by Brennan et al, 1995) At both ages, animals are checked daily for any disruption of the skin edges and any remaining sutures are removed at 5 days. Animals were kept warm during recovery from anaesthesia, and returned to their dam or home cage as soon as possible.

**Behavioural testing**

Rat pups were placed on a warming blanket and hand held calibrated von Frey filaments (0.13g to 7.8g) were sequentially applied to the dorsum of the hindpaw. This allows more reliable application of von Frey hair stimuli to the small paw as testing on an elevated mesh platform at this age often causes lifting of the paw rather than a clear withdrawal response and it is difficult to maintain body temperature. We have previously shown similar values with testing on the dorsum and plantar surfaces.,^2^ and this methodology is sufficiently sensitive to identify hyperalgesia following incision and hindpaw inflammation, and also dose-dependent analgesic effects.^4, 5^ The maximum force applied was that which evoked five withdrawal responses. A sigmoidal stimulus-response curve was generated for each animal with the midpoint (50% effective force, EF_50_), calculated as the threshold.^2, 6^

At 6 weeks of age, young adult rats were habituated to the test apparatus, and then mechanical withdrawal threshold and thermal withdrawal latency were measured at baseline and at regular intervals to 21 days following adult incision. For mechanical withdrawal, animals were placed on an elevated mesh platform in individual opaque plexiglass chambers, and a mechanical stimulus (electronic von Frey device with ramp 20g/s and maximum 50g; Dynamic Plantar Aesthesiometer, Ugo Basile) was applied to the plantar surface of the hindpaw. The threshold was defined as the mean of three measures of the force evoking a brisk withdrawal response. For thermal latency, animals were habituated to the heated glass surface of a modified Hargreaves apparatus (University Anesthesia Research and Development Group, University of California San Diego, La Jolla, CA), the time for withdrawal from a heat stimulus directed at the mid-plantar paw was recorded, and the mean of three measures was calculated.^3^

**Electromyography (EMG) recordings**

Animals were anaesthetised with isoflurane (2-4%) in oxygen, and a tracheal tube was inserted for mechanical ventilation (pressure limited time cycled Small Animal Ventilator, Harvard Apparatus Ltd). Animals were supported in a spinal frame with the left hindpaw secured on a fixed platform. Isoflurane was reduced to 1.2% in oxygen for 20 minutes to allow equilibration to a stable plane of anaesthesia that was maintained during EMG recordings. This depth was sufficient to allow mechanical ventilation without requiring a muscle relaxant, but did not excessively suppress reflex sensitivity. Body temperature was measured and maintained with a rectal probe connected to a thermostatically controlled heat-pad. Heart rate was continuously monitored with an electrocardiogram (Vetronics ERM-8010 ECG; Vetronic Services, Devon, UK), and in later experiments, continuous heart rate and oxygen saturation were monitored (MouseOx® Starr Life Sciences Corp., PA, USA).

A bipolar EMG electrode (Ainsworks, London) comprising a stainless steel 30G needle with a central copper wire core was placed through a small skin incision into the biceps femoris muscle. Von Frey hairs were applied to the plantar surface of the hindpaw for 1 second and the EMG response to the mechanical stimulus was processed (Neurolog, Digitimer, Hertfordshire, UK) and recorded in 12-second epochs (PowerLab 4S, AD Instruments, Castle Hill, Australia). Von Frey hairs with logarithmically increasing bending force from 13 to a maximum of 120 grams (vFh number 14 to 20) were sequentially applied to the hindpaw, with at least 60 seconds between stimuli. The duration of the EMG response was outlined from the display of the raw data and the integral of the root mean square (RMS) of the signal calculated (EMG response)(Chart, Powerlab AD Instruments). The EMG response was plotted against the von Frey hair number (mechanical stimulus) and the area under the stimulus-response curve (AUC) calculated to quantify the overall “reflex response” ^2, 3^.

**Drug administration and dose-finding pilot experiments**

Morphine dose-dependently increases mechanical withdrawal threshold, but doses vary with age and route of administration.^7, 8^ In pilot experiments we established intrathecal (IT) and subcutaneous (sc) doses that would: i) produce a similar change in mechanical withdrawal threshold and demonstrate accurate drug delivery prior to incision, and ii) maintain the threshold at or above baseline (i.e. anti-hyperalgesic effect) for the 2 hour interval between injections. Midline percutaneous IT injections were performed between the lower lumbar vertebrae with morphine 0.1mg kg^-1^ or saline in an injectate volume of 0.5mcl g^-1^ to produce lumbar and low thoracic intrathecal spread as previously described.^7^ Subcutaneous (sc) injection of 1mg kg^-1^ morphine (5mcl g^-1^ of 0.2mg ml^-1^) was performed in the same midlumbar region to ensure the behavioural tester was blind to route of injection.

The effect of morphine on EMG measures of reflex sensitivity was assessed in anaesthetized adult animals. In pilot experiments, baseline EMG responses were quantified in animals with or without plantar incision 24 hours previously, and 15 and 30 minutes following s.c. morphine (0.5-1mg kg^-1^) injection in the contralateral hindlimb. Morphine 1mg kg^-1^ sc markedly reduced reflex sensitivity, with minimal residual reflex response to the maximum von Frey hair stimulus in incised animals, and loss of reflex response to noxious pinch in naïve animals. Morphine 0.5mg/kg reduced reflex sensitivity in naïve animals but produced minimal change in incised animals. Following morphine 0.75mg kg^-1^, a response to noxious pinch was maintained in 7 of 8 non-incised animals, there was a quantifiable reduction in reflex response in incised animals (1.5mcl g^-1^ of 0.5mcg mcl^-1^ or 0.5mg ml^-1^ solution), and similar effects were seen at 15 and 30 minutes. Therefore, in all included experiments, EMG responses to graded mechanical stimuli on the left hindpaw were recorded (EMG AUC_1)_, 0.75mg kg^-1^ morphine was then administered subcutaneously in the right thigh, and a second run of EMG recordings was commenced 15 minutes later (EMG AUC_2_). The response to morphine was calculated as the percentage change: [(EMG AUC_2_ / EMG AUC1) x 100].

Sciatic nerve blockade was performed in P3 pups using the same technique, local anaesthetic dose and frequency of injection as our previous experiments.^2, 6^ Percutaneous injections of 40mcl of 0.5% levobupivacaine (Chirocaine 50mg/10ml; Abbott Laboratories Limited, Maidenhead, Berkshire, United Kingdom) were performed during brief anaesthesia. As sciatic blockade is relatively short-lived in rat pups, a pre-operative block plus a further 2 injections at 2-hourly intervals were performed to maintain afferent blockade during the early peri-operative period.

**Conditioned place preference**

Conditioned place preference (CPP) aims to capture the aversive dimension of pain by assessing motivational drive to avoid pain or seek relief from ongoing or spontaneous pain.^9^ Morphine-induced CPP has been extensively described in the literature, though doses and conditioning paradigms differ significantly across studies. Single-trial conditioning paradigms (similar to that used in the present study) have been described both in the presence^10, 11^ and absence^12, 13^ of pain stimuli, with place preference induced by doses of 0.5-8mg kg^-1^ administered by sc, intraperitoneal or intravenous routes. Morphine 2.5mg kg^-1^ was not associated with any significant place preference in sham animals.^11^ Inflammatory pain (CFA) facilitated morphine-induced CPP, with fewer conditioning sessions required to produce a significant place preference.^14^ Single-trial conditioning was demonstrated 24 hours following plantar hindpaw incision with preference for the analgesia-paired chamber shown following popliteal fossa injection of lidocaine^15^ or sciatic nerve block with bupivacaine.^16^

Experiments were performed in adult male rats following neonatal incision and/or subcutaneous morphine. P3 rat pups were randomly allocated to subcutaneous injection of 1mg kg^-1^ morphine (5mcl g^-1^ of 0.2mg ml^-1^) or saline in the midlumbar region, with 3 injections at 2 hourly intervals starting 30 mins prior to incision. An additional group received morphine alone. At 6-7 weeks age, neonatal intervention groups, age-matched naïve and adult-only-incision groups underwent CPP testing as previously described.^16^ The apparatus comprised two large end chambers (20 x 18 x 25 cm) with distinct visual cues (black spots or grey stripes) and a smaller connecting chamber (20 x 7 x 25 cm). On the preconditioning day, animals were placed in the central connecting chamber with free access to all three chambers for a period of 15 min. The time spent in each chamber was recorded and the “preferred” and “non-preferred” end-chambers were identified. In the afternoon, animals underwent plantar incision. Single-trial conditioning using a biased design was performed the following day. In the morning of the conditioning day, all animals received subcutaneous saline (1ml kg^-1^) and were placed in the preferred chamber for 45 min, with no access to the other chambers. In the afternoon (approximately 4 hours later), rats received subcutaneous morphine (2mg kg^-1^) and were placed in the non-preferred chamber, with no access to the other chambers. The following day (2 days post-incision), the animals were placed back in the central chamber with free access to all chambers, and the time spent in each chamber over a period of 15 min was measured.

CPP data are expressed as an absolute measure of preference (total time spent in the initially non-preferred chamber during pre-conditioning vs. during the test session) or a relative measure of preference (difference score).^16^ The difference score was calculated as the time spent in a chamber during the test session minus the time spent in that chamber during pre-conditioning, and therefore, a positive difference score for the initially non-preferred chamber indicates the development of a preference for the morphine-paired chamber.

**Novel object recognition**

The novel object recognition (NOR) test was used to assess recognition memory,^17^ with methodology similar to previous descriptions by the current experimenter.^18^ Rat pups at P3 were randomly allocated to neonatal incision or anaesthesia-only treatments groups, followed by NOR testing at 6-8 weeks (males; 295-320g, females; 210-230g), and comparison to age-matched naïve and adult-only-incision groups. Testing was carried out in a grey plastic arena (90 x 90 x 40 cm), with a video camera positioned above to record behaviour. Objects with no apparent natural significance (object a and b: weighted plastic bottles; novel object: abstract structure made from coloured plastic toy blocks) were secured to the base of the arena, 30 cm from the sides of opposite corners. Animals were habituated to the arena in the absence of objects for 30 min two days before the test day, and adult incisions were performed on the following day. The test day comprised three stages: i) habituation; ii) exposure 1; and iii) exposure 2. Rats were introduced to the arena for a three-minute habituation period and then returned to their home cage for seven minutes. During exposure 1, two identical objects were placed, and rats allowed to freely explore the arena and objects for a period of 3 min, then returned to their home cage for an interval of 10 min. For exposure 2, one of the objects was replaced with a novel object, and rats freely explored the arena and objects for a period of 3 min before return to their home cage. Animal movement was recorded with Ethovision® behavioural tracking software (Noldus, Netherlands). Videos were reviewed and the duration of object exploration (defined as sniffing, rearing against, or having the head directed towards the object) within an annulus of approximately 2 cm was manually timed by an experimenter blinded to treatment. The proportion of time spent exploring the object was assessed by calculating a discrimination ratio as total time spent exploring either object divided by total time spent exploring both objects. Tracking of locomotor activity confirmed no between-group differences in motor activity.

**Tissue Analysis**

Rats were terminally anaesthetised with pentobarbital (i.p. 100mg/kg, Euthetal, Merial Animal Health Ltd., UK) and transcardially perfused with heparinised saline followed by 4% paraformaldehyde (Fisher Scientific, UK). Spinal cords were exposed, and the sciatic nerve spinal roots traced back to identify the L4/L5 spinal segments. Tissue was post-fixed in 4% paraformaldehyde, cryoprotected in sucrose (30% sucrose, 0.02% sodium azide in 0.1M phosphate buffer) and stored at 4°C prior to sectioning.

*cFos immunohistochemistry*

P3 rats received morphine (IT or s.c.), saline, or sciatic block with local anaesthetic 30 min before plantar hindpaw incision, and a second dose 90 min post incision. Spinal cords were removed two hours following incision. Transverse 40μm sections were cut with a microtome (Leica, Germany), and sequential sections free-floated in wells of 5% sucrose solution in 0.1 M phosphate buffer (PB). Sections were washed in 0.1M phosphate buffer prior to and between the following steps: i) block solution (3% goat serum, 3% TritonX, and 2% hydrogen peroxide in PB) for 1 hour at room temperature; ii) rabbit anti-c-Fos antibody (Calbiochem, USA) 1:5000 in TTBS overnight at room temperature; iii) biotinylated secondary antibody solution at 1:500 in TTBS (goat anti-rabbit, Vector Laboratories, USA) for 2 hours at room temperature; and iv) Avidin-Biotin Complex (ABC) solution (Vector Laboratories, USA) for 1 hour at room temperature. Colour was developed by incubation with 3-3’-Diaminobenzidine (DAB) Peroxidase substrate (DAB staining kit, Vector Laboratories, USA). Sections were mounted on slides (Superfrost Plus Microscope Slides, VWR, USA), left to dry overnight, dehydrated in a series of ascending concentrations of ethanol, cleared in Histo-Clear (National Diagnostics, USA), and coverslipped with DPX mounting medium (Sigma-Aldrich, USA).

Images were obtained with a Leica DMR microscope (Leica, Germany) and ORCA-100 C4742-95 digital camera (Hamamatsu, Japan) and collected on Volocity software (Volocity, USA). Images of whole sections were taken at a 10x magnification on a Bright Field setting. The number of Fos-like immunoreactive cells was counted in laminae I/II, II/IV, and V with the aid of the ImageJ Cell Counter plugin (NIH, USA). Laminae were defined with reference to a neonatal rat spinal cord atlas. Counts for each animal were averaged from a minimum of 3 sections.

*Ionized calcium-binding adapter molecule 1 (Iba1) immunohistochemistry*

Following morphine (IT or sc), saline, or sciatic block with local anaesthetic before and after plantar hindpaw incision on P3, rats were terminally anaesthetized and spinal cords removed on P6. Transverse 20µm sections were cut on a cryostat (Leica, Germany), and sequential sections free-floated in wells of 5% sucrose solution in 0.1 M phosphate buffer prior to mounting on Superfrost Plus slides. Slides were washed initially and between subsequent steps with phosphate-buffered saline (PBS) containing 0.1% Triton X-100, blocked for 1h at room temperature (5% chicken serum in PBS), and then incubated for 24h with primary goat anti-Iba1 antibody (1:400, AbCam, UK) followed by AlexaFluor® 594-conjugated chicken anti-goat IgG (1:200, Invitrogen, USA) for 24h at room temperature. Sections were cover-slipped with Prolong Gold fluorescent mounting media (Molecular Probes, USA). Images were obtained under a TRITC filter at a 10x magnification. Within a fixed size region of interest, Iba1-IR cells were counted in the ipsilateral and contralateral medial superficial dorsal horn. Counts for each animal were averaged from a minimum of 4 sections.

*Mu opioid receptor (MOR) immunohistochemistry*

Twenty micron sections of lumbar spinal cord were cut (Leica CM 1800 cryostat, San Marcos, CA), and mounted on Superfrost Plus slides (Fischer Scientific, Houston, Texas). Washes with 0.1M phosphate buffered saline (PBS) were performed prior to, and between, subsequent steps. Sections were incubated with: 10% normal goat serum (Vector Laboratories) plus 0.3% triton X (Sigma-Aldrich) in PBS for 2 hours at room temperature; polyclonal rabbit anti-MOR (1:500; Neuromics Inc.) and mouse anti-neuronal nuclear antigen (NeuN; 1:500; Chemicon International®) in 3% normal goat serum 0.3% triton X and 0.1M PBS for 48 hours at 4^0^C; and finally secondary antibodies (1:200 goat anti-rabbit Alexa Fluor 488 and 1:200 goat anti-mouse Alexa Fluor 594; Invitrogen) for 2 hours at room temperature. Additional sections were labelled with NeuN, MOR antibodies and isolectin B4 (IB4; 1:500; Chemicon International®). Slides were cover slipped with Fluoromount (Sigma-Aldrich).

**Experimental Timeline (see also Supplementary Table 1)**

On postnatal day 3, litters of pups were separated into males and females, reduced to a maximum of 12 pups, weighed, and then randomly numbered and allocated to treatment groups (equal numbers of males and females). Following measurement of mechanical withdrawal threshold, injections were performed under brief anaesthesia (saline, IT morphine, sc morphine, or sciatic nerve LA block) and thresholds measured again at 20 minutes to confirm drug effect (increase in threshold following morphine; motor and sensory block following sciatic LA) prior to plantar incision. Mechanical withdrawal threshold was measured before and after 2 further injections at 2-hourly intervals. Naïve animals underwent the same degree of maternal separation and handling only, and an additional group received subcutaneous morphine only. Animals were returned to the dam between interventions, and to the home cage at the end of the procedures. The following day, body weight and mechanical threshold were measured. Additional groups for tissue analysis were terminally anaesthetized and spinal cords removed at 2 hours, 3 days or 6 weeks for c-fos, Iba1 or morphine opioid receptor immunohistochemistry.

At 6 weeks of age, animals had body weight, mechanical withdrawal threshold and thermal withdrawal latency measured prior to adult incision. Treatment groups included: naïve; IN (adult only incision); nsIN-IN (neonatal saline and repeat incision); nIN(IT)-IN (neonatal intrathecal morphine and repeat incision); nIN(sc)-IN (neonatal subcutaneous morphine and repeat incision); nIN(LA)-IN (neonatal sciatic block and repeat incision); n(sc)-IN (neonatal subcutaneous morphine and adult incision only). Groups were assigned to the following experimental outcomes: thermal and mechanical behavioural thresholds to 21 days following adult incision; EMG recordings of reflex sensitivity 24 hours post-incision; conditioned place preference to morphine-paired chamber; EMG recording 24 hours post incision and percentage reduction in reflex sensitivity by subcutaneous morphine 0.75mg kg^-1^; or novel object recognition.

**References**

1 Brennan TJ, Vandermeulen EP, Gebhart GF. Characterization of a rat model of incisional pain. *Pain* 1996; **64**: 493-501

2 Walker SM, Tochiki KK, Fitzgerald M. Hindpaw incision in early life increases the hyperalgesic response to repeat surgical injury: critical period and dependence on initial afferent activity. *Pain* 2009; **147**: 99-106

3 Beggs S, Currie G, Salter MW, Fitzgerald M, Walker SM. Priming of adult pain responses by neonatal pain experience: maintenance by central neuroimmune activity. *Brain* 2012; **135**: 404-17

4 Walker SM, Howard RF, Keay KA, Fitzgerald M. Developmental age influences the effect of epidural dexmedetomidine on inflammatory hyperalgesia in rat pups. *Anesthesiology* 2005; **102**: 1226-34

5 Walker SM, Grafe M, Yaksh TL. Intrathecal clonidine in the neonatal rat: dose-dependent analgesia and evaluation of spinal apoptosis and toxicity. *Anesth Analg* 2012; **115**: 450-60

6 Walker SM, Fitzgerald M, Hathway GJ. Surgical injury in the neonatal rat alters the adult pattern of descending modulation from the rostroventral medulla. *Anesthesiology* 2015; **122**: 1391-400

7 Westin BD, Walker SM, Deumens R, Grafe M, Yaksh TL. Validation of a Preclinical Spinal Safety Model: Effects of Intrathecal Morphine in the Neonatal Rat. *Anesthesiology* 2010; **113**: 183-99

8 Nandi R, Beacham D, Middleton J, Koltzenburg M, Howard RF, Fitzgerald M. The functional expression of mu opioid receptors on sensory neurons is developmentally regulated; morphine analgesia is less selective in the neonate. *Pain* 2004; **111**: 38-50

9 Navratilova E, Xie JY, King T, Porreca F. Evaluation of reward from pain relief. *Ann N Y Acad Sci* 2013; **1282**: 1-11

10 Cahill CM, Xue L, Grenier P, Magnussen C, Lecour S, Olmstead MC. Changes in morphine reward in a model of neuropathic pain. *Behav Pharmacol* 2013; **24**: 207-13

11 Hung CH, Wang JC, Strichartz GR. Spontaneous Chronic Pain After Experimental Thoracotomy Revealed by Conditioned Place Preference: Morphine Differentiates Tactile Evoked Pain From Spontaneous Pain. *J Pain* 2015; **16**: 903-12

12 Bardo MT, Neisewander JL. Single-trial conditioned place preference using intravenous morphine. *Pharmacol Biochem Behav* 1986; **25**: 1101-5

13 Fenu S, Spina L, Rivas E, Longoni R, Di Chiara G. Morphine-conditioned single-trial place preference: role of nucleus accumbens shell dopamine receptors in acquisition, but not expression. *Psychopharmacology (Berl)* 2006; **187**: 143-53

14 Zhang Z, Tao W, Hou YY, Wang W, Lu YG, Pan ZZ. Persistent pain facilitates response to morphine reward by downregulation of central amygdala GABAergic function. *Neuropsychopharmacology* 2014; **39**: 2263-71

15 Navratilova E, Xie JY, Okun A, et al. Pain relief produces negative reinforcement through activation of mesolimbic reward-valuation circuitry. *Proc Natl Acad Sci U S A* 2012; **109**: 20709-13

16 Dalm BD, Reddy CG, Howard MA, Kang S, Brennan TJ. Conditioned place preference and spontaneous dorsal horn neuron activity in chronic constriction injury model in rats. *Pain* 2015; **156**: 2562-71

17 Ennaceur A, Delacour J. A new one-trial test for neurobiological studies of memory in rats. 1: Behavioral data. *Behav Brain Res* 1988; **31**: 47-59

18 Moriarty O, Gorman CL, McGowan F, et al. Impaired recognition memory and cognitive flexibility in the rat L5-L6 spinal nerve ligation model of neuropathic pain. *Scand J Pain* 2016; **10**: 61-7
