# Supplementary material for: Opioid analgesia and the somatosensory memory of neonatal surgical injury in the adult rat
Source: Br J Anaesth. 2018 Feb 1;121(1):314–24. doi: 10.1016/j.bja.2017.11.111 (PMC6200106; doi:10.1016/j.bja.2017.11.111)
Supplement: mmc2 [file mmc2.docx]

**Supplementary Table 1. Experimental Timelines, Group Allocation and Outcomes.**

**A:** Neonatal and Adult Groups with Interventions designated as “+”

| **NEONATAL GROUP** | **NEONATAL DRUG**  (3 x 2hrly: 30 mins pre-incision, 90 & 150 mins post-incision) | **NEONATAL**  **INCISION**  **(P 3)** | **→** | **ADULT**  **INCISION**  **(6 weeks)** | **ADULT GROUP** |
| --- | --- | --- | --- | --- | --- |
| **naive** | **-** | **-** | **Return to BSU**  **Wean at 3wks age** | **-** | **naive** |
| **naive** | **-** | **-** |  | **+** | **IN** |
| **nsIN** | saline | **+** |  | **+** | **nsIN-IN** |
| **nIN(IT)** | intrathecal morphine 0.1mg kg^-1^ | **+** |  | **+** | **nIN(IT)-IN** |
| **nIN(sc)** | subcutaneous morphine 1mg kg^-1^ | **+** |  | **+** | **nIN(sc)-IN** |
| **nIN(LA)** | sciatic block 40 mcl levobupivacaine | **+** |  | **+** | **nIN(LA)-IN** |
| **n(sc)** | subcutaneous morphine 1mg | **-** |  | **+** | **n(sc)-IN** |

**B:** Neonatal and Adult Outcomes with allocated groups designated as “+”

| **OUTCOMES IN NEONATAL RAT** | | | | | |  | **OUTCOMES IN YOUNG ADULT RAT** | | | | | | |
| --- | --- | --- | --- | --- | --- | --- | --- | --- | --- | --- | --- | --- | --- |
| **GROUP** | **EXPT D**  Neonatal response to incision and morphine | | Tissue Analysis  Supplementary Data | | |  | **GROUP** | **EXPT A**  Re-incision hyperalgesia | **EXPT B**  Neonatal morphine vs LA positive control | | **EXPT C**  Neonatal intervention and adult response to opioid | | |
|  | Behavioural hyperalgesia  (Fig 4A) | 24 hr threshold  (Fig 4B) | c-fos  (Fig S2) | Iba1  (Fig S2) | MOR  (Fig S3) |  |  | Behavioural  Hyperalgesia(0-21 days) (Fig 1;Fig S1) | Baseline threshold  (Fig 2B) | EMG reflex sensitivity  (Fig 2A) | EMG post morphine  (Fig 3C) | CPP to morphine  (Fig 3A,B) | NOR  (Fig 3D) |
| **naive** | **-** | **+** [*n*=11] | **+** [*n*=6] | **+** [*n*=8] | **+** (P3,40) |  | **naive** | **-** | **-** | **+** [*n*=10] | **+** [*n*=10] | **+** [*n*=8] | **+** [*n*=14] |
|  | | | | | |  | **IN** | **+** [*n*=8] | **+** [*n*=13] | **+** [*n*=13] | **+** [*n*=12] | **+** [*n*=12] | **+** [*n*=6] |
| **nsIN** | **+** [*n*=12] | **+** [*n*=12] | **+** [*n*=10] | **+** [*n*=10] | **-** |  | **nsIN-IN** | **+** [*n*=8] | **+** [*n*=13] | **+** [*n*=13] | **+** [*n*=13] | **+** [*n*=12] | **+** [*n*=20] |
| **nIN (IT)** | **+** [*n*=12] | **+** [*n*=12] | **+** [*n*=10] | **+** [*n*=6] | **-** |  | **nIN(IT)-IN** | **+** [*n*=8] | **+** [*n*=13] | **+** [*n*=11] | **-** | **-** | **-** |
| **nIN (sc)** | **+** [*n*=12] | **+** [*n*=12] | **+** [*n*=10] | **+** [*n*=10] | **-** |  | **nIN(sc)-IN** | **+** [*n*=8] | **+** [*n*=13] | **+** [*n*=13] | **+** [*n*=13] | **+** [*n*=12] | **-** |
| **nIN(LA)** | **-** | **+** [*n*=12] | **+** [*n*=6] | **+** [*n*=6] | **-** |  | **nIN(LA)-IN** | **-** | **+** [*n*=10] | **+** [*n*=10] | **-** | **-** | **-** |
| **n(sc)** | **-** | **-** | **-** | **-** | **+** (P40) |  | **n(sc)-IN** | **-** | **-** | **-** | **+** [*n*=10] | **+** [*n*=9] | **-** |
|  | Total: *n*=59 | | Tot:*n*=42 | Tot:*n*=40 | Tot:*n*=12 |  |  | Total: *n*=32 | Total: *n*=82 | | | Total: *n*=53 | Tot: *n*=40 |

*Legend:* P, postnatal day; s, saline; IN, incision; IT, intrathecal; sc, subcutaneous; LA, local anaesthetic; BSU, Biological Services Unit; MOR, morphine opioid receptor; EMG, electromyography; CPP, conditioned place preference; NOR, novel object recognition
