# Supplementary material for: Opioid analgesia and the somatosensory memory of neonatal surgical injury in the adult rat
Source: Br J Anaesth. 2018 Feb 1;121(1):314–24. doi: 10.1016/j.bja.2017.11.111 (PMC6200106; doi:10.1016/j.bja.2017.11.111)
Supplement: mmc3 [file mmc3.pdf]

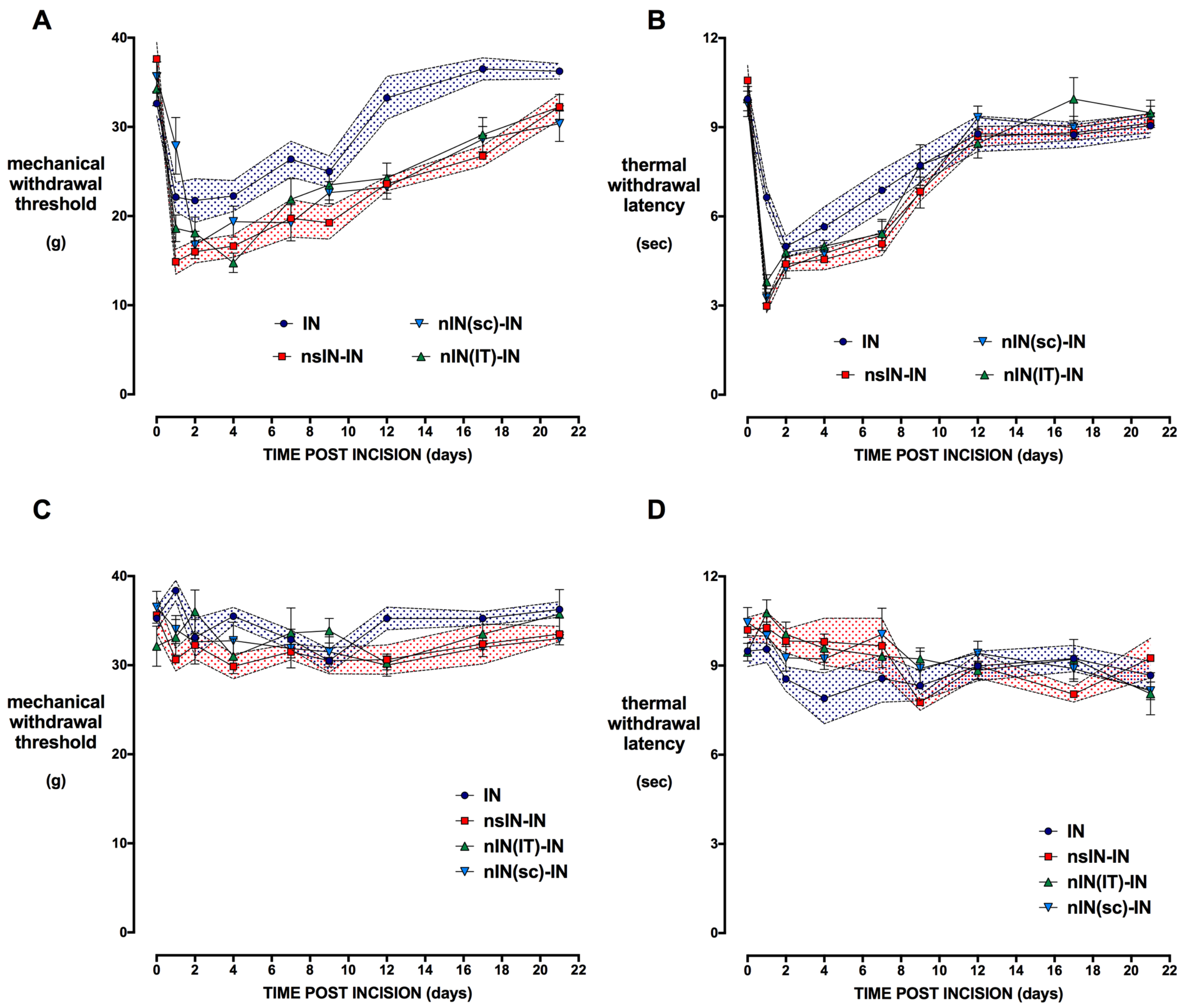

Supplementary Fig. 1. Raw data confirm group differences in absolute sensory threshold values over time in the incised but not contralateral paws.

A: Mechanical withdrawal threshold at baseline and at time points to 21 days post incision show main effect of group ($F_{3,28}$=6.9, P=0.02) and time ($F_{8,224}$=63.9, P<0.01) with significant differences between IN vs nsIN-IN (P<0.01) and IN vs nIN(IT)-IN (P<0.05). Both the higher baseline threshold and the lower threshold values contribute to the greater degree of change in the nsIN-IN vs IN groups. Data=mean±SEM. SEM bands demonstrate clear differences between IN and nsIN-IN groups in both the degree and duration of mechanical hyperalgesia; *n*=8 per group.

B: Thermal withdrawal latency raw data shows a main effect of group ($F_{3,28}$=3.3, P=0.03) and time ($F_{8,224}$=2.1, P<0.01) with significant differences between IN vs nsIN-IN (P=0.046).

C, D: Control contralateral paw values for mechanical threshold (C) or thermal latency (D) do not change with time.
