# Supplementary material for: Opioid analgesia and the somatosensory memory of neonatal surgical injury in the adult rat
Source: Br J Anaesth. 2018 Feb 1;121(1):314–24. doi: 10.1016/j.bja.2017.11.111 (PMC6200106; doi:10.1016/j.bja.2017.11.111)
Supplement: mmc4 [file mmc4.pdf]

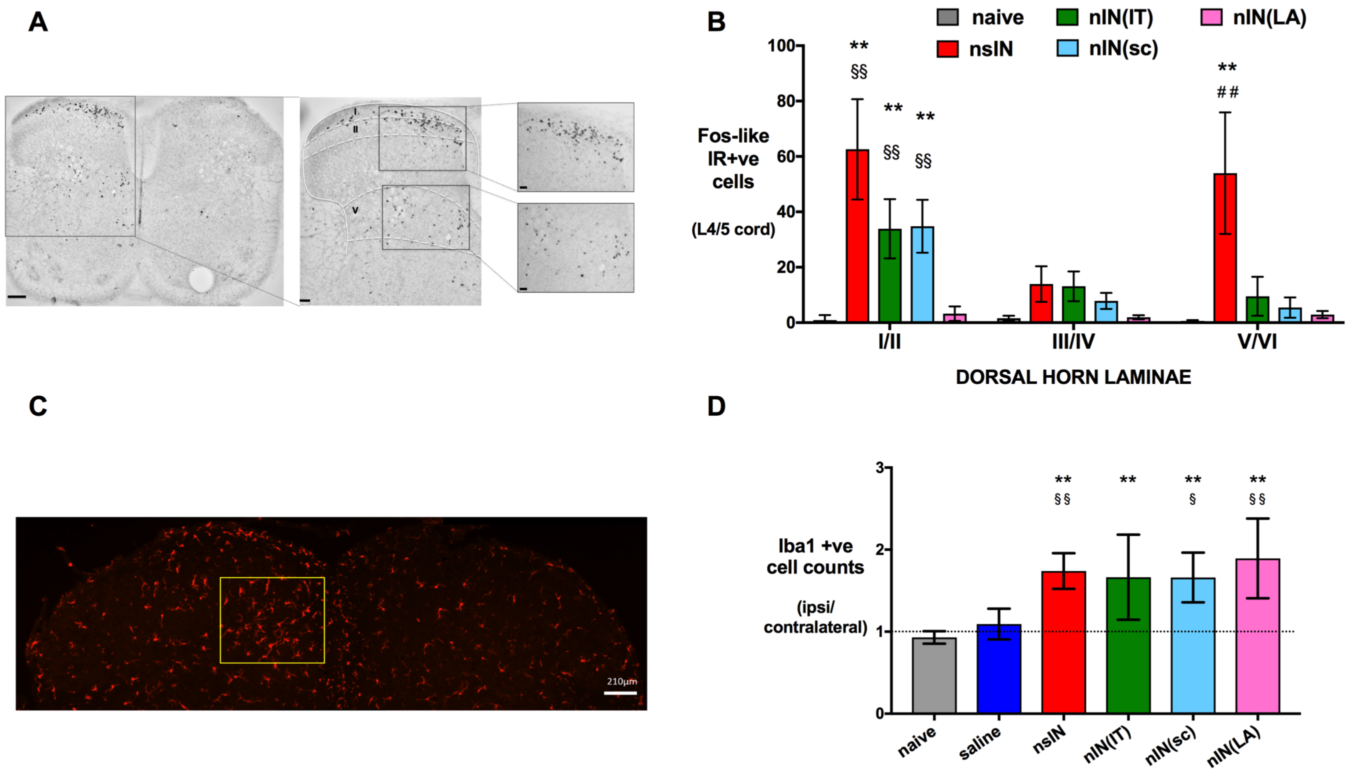

Supplementary Fig 2. Acute impact of neonatal incision, with or without analgesia, on spinal neuronal activation and microglial reactivity.

A: Representative lumbar L4,5 spinal segment with Fos-like immunoreactivity two hours following P3 plantar hindpaw incision, with magnification of superficial (I/II) and deep (V) laminae (left = ipsilateral; scale bars bottom left = 26micron). The number of Fos-like immunoreactive cells was counted in laminae I/II, II/IV, and V with the aid of the ImageJ Cell Counter plugin (NIH, USA). Laminae were defined with reference to a neonatal rat spinal cord atlas. Counts for each animal were averaged from a minimum of 3 sections.

B: Fos-like immunoreactive (IR+ve) cell counts in laminae I/II are increased by incision and reduced by morphine but not to same degree as sciatic block (***P* <0.001 vs naïve; §§ *P* <0.01 vs sciatic block). In laminae V, counts are increased by incision and all analgesia groups do not differ from naive (***P* <0.001 vs naïve; ## *P* ≤0.01 IN vs all analgesia groups); two-way ANOVA with Tukey *post-hoc* comparisons. Bars=mean [95%CI]; *n* = number of animals = 6-10 per group.

C: Representative lumbar L4,5 dorsal spinal cord section with Iba1 immunohistochemistry 3 days following plantar incision on P3 (left = ipsilateral to incision). Yellow square represents fixed size region of interest (ROI) for counts in medial superficial dorsal horn. Scale bar = 210 micron.

D: Iba1 immunoreactive(Iba1 +ve) cell counts in spinal dorsal horn ROI 3 days following incision expressed as the ratio of ipsilateral over contralateral. Three days following neonatal incision, microglial cell counts were significantly increased in the ipsilateral medial superficial DH (mean± SD; ipsilateral 15.1±3.0 vs contralateral 8.8±0.5) and were also higher than age-matched naïve (6.6±0.7) animals. To allow within

animal comparison in the same spinal segments, the ipsilateral versus contralateral ratio of cell counts was calculated and showed a main effect of group ($F_{5,40}$=8.2, P<0.0001) but not sex ($F_{1,40}$=0.11 P=0.75). Male and female data are combined. Peri-incision morphine or sciatic nerve block did not prevent the ipsilateral dorsal horn increase in microglial cell counts 3 days following neonatal incision. **P<0.01 vs naïve; §P<0.05, §§P<0.01 vs saline. Points=individual data, bars = mean [95%CI]; one way ANOVA with Tukey's post-hoc comparisons.
