# Supplementary material for: Opioid analgesia and the somatosensory memory of neonatal surgical injury in the adult rat
Source: Br J Anaesth. 2018 Feb 1;121(1):314–24. doi: 10.1016/j.bja.2017.11.111 (PMC6200106; doi:10.1016/j.bja.2017.11.111)
Supplement: mmc5 [file mmc5.pdf]

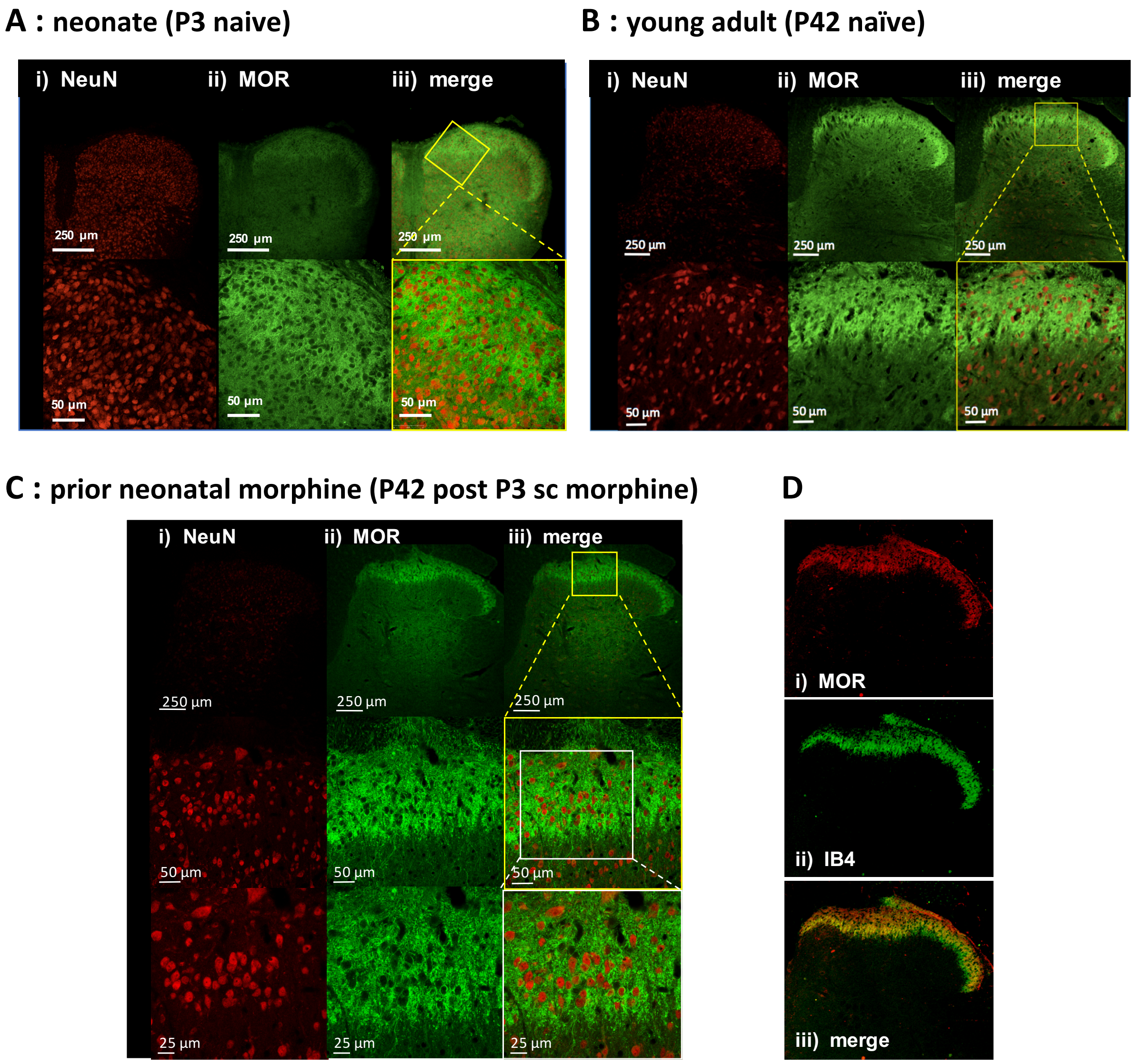

Supplementary Fig 3. Expression of mu opioid receptor (MOR) in lumbar spinal dorsal horn changes with postnatal age but not prior neonatal morphine.

A,B: Representative spinal dorsal horn sections double-labelled with antibodies to the neuronal marker NeuN (red) and mu opioid receptor (MOR; green) in naïve animals. At P3, MOR expression extends more broadly through the dorsal horn (A), but is restricted to the superficial laminae by P42 (B). Note: relative size of the 250 and 50 µm scale bar is greater in the smaller cord at P3.

C,D: Prior administration of systemic morphine (1mg/kg x3 2-h doses) at P3 does not alter the laminar distribution of MOR-immunoreactivity (C), and restriction to the superficial laminae is highlighted by the relationship to Isolectin B4 (IB4) in lamina II inner (D).
